# A pragmatic risk-stratified framework for using large language models in intensive care medicine: A narrative review

**DOI:** 10.1016/j.ccrj.2026.100194

**Published:** 2026-06-25

**Authors:** Nilesh Anand Devanand, Santosh Verghese, Stephen Bacchi, Christopher Bain, Ashwin Subramaniam, Krishnaswamy Sundararajan

**Affiliations:** a Department of Intensive Care, Royal Adelaide Hospital, Port Road, Adelaide, South Australia, Australia; b Department of Intensive Care, Flinders Medical Centre, Southern Adelaide Local Health Network, Adelaide, South Australia, Australia; c Office of the Chief Medical Information Officer, Digital Health, Adelaide, South Australia; d Department of Neurology, Lyell McEwin Hospital, Haydown Road, Elizabeth Vale, South Australia, Australia; e Department of Digital Health, Faculty of Information Technology, Monash University, Australia; f Department of Intensive Care Medicine, Monash Health, Dandenong, Victoria, Australia; g Department of Intensive Care, Peninsula Health, Frankston, Victoria, Australia; h Peninsula Clinical School, Monash University, Frankston, Victoria, Australia; i Australian and New Zealand Intensive Care Research Centre, Department of Epidemiology and Preventive Medicine, Monash University, Melbourne, Victoria, Australia; j School of Medicine, The University of Adelaide, Adelaide, South Australia, Australia

**Keywords:** Large language model, Intensive care unit, Clinical decision support, Artificial intelligence, Health governance

## Abstract

**Objective:**

To provide Australian intensive care clinicians with a pragmatic framework for the safe integration of large language models (LLMs) into intensive care unit (ICU) practise, addressing the current lack of Australian-specific guidance and limited local evidence.

**Design:**

Narrative review.

**Data sources:**

Peer-reviewed publications, preprints, and policy documents relating to LLM use in health care, with a focus on critical care applications and governance.

**Review methods:**

Evidence and expert commentary were synthesised to develop a clinician-led, risk-stratified framework for ICU implementation, with emphasis on safety, oversight, and applicability within Australian health systems. Clinical use cases, risks, governance considerations, and practical safeguards for day-to-day ICU practise were identified.

**Results:**

LLMs have potential utility in data-dense ICU environments, including summarising complex clinical information, supporting documentation, assisting clinical reasoning, and facilitating research tasks. However, evidence for LLM performance in ICU contexts remains limited, particularly in Australia. Key risks include inaccurate or fabricated outputs (“*hallucinations*”), bias, lack of situational awareness, privacy concerns, and over-reliance in high-stakes decision-making. We propose a risk-stratified framework that categorises LLM applications by clinical risk and reversibility, and aligns each category with proportional oversight, verification processes, and governance safeguards, emphasising clinician-in-the-loop decision-making.

**Conclusions:**

LLMs may serve as adjunctive cognitive tools in Australian ICUs when used in clearly defined, low-to intermediate-risk contexts under clinician oversight. Safe integration requires robust governance frameworks emphasising transparency, data protection, and proportionate clinician decision-making. Further Australian-based evaluation is needed before high-risk clinical applications can be considered for routine practise.

## Graphical abstract

**Figure:**
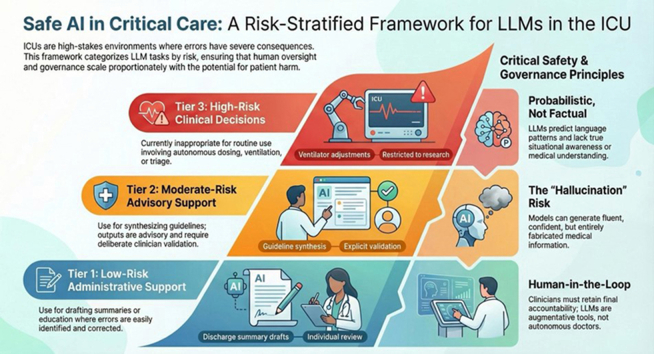

## 1 Introduction

Artificial intelligence (AI) has been increasingly integrated into medicine and clinical practise in recent years, supporting tasks such as pattern recognition, decision support, and information synthesis.^1^^,^^2^ Early AI systems were limited to well-defined tasks and rule-based functions, but advances in machine learning and neural networks have enabled the development of large language models (LLMs), which are trained on large-scale text and code datasets to generate and summarise human language.[3], [4], [5]

In healthcare, LLM applications now extend beyond administrative documentation to include clinical decision support, education, and evidence synthesis, with early evaluations demonstrating both promising performance and significant limitations in clinical reasoning tasks.[6], [7], [8] These capabilities are particularly relevant to the intensive care unit (ICU), a data-rich environment characterised by time-critical decision-making, clinical uncertainty, and high consequences of error. However, the same characteristics that make the ICU attractive for LLM deployment also amplify potential risks. Errors, bias, and hallucinated outputs introduce significant patient safety risks in these high-stakes settings.[9], [10], [11] Over-reliance on automated text generation may compound these risks, with potential medicolegal consequences.^10^^,^^12^

Despite rapidly growing interest in generative AI, much of the existing literature remains technology-focused, descriptive, or policy-oriented, with limited operational guidance for clinicians.^13^^,^^14^ In particular, recent discussions of generative AI in Australian healthcare have largely addressed opportunities, risks, and high-level ethical considerations, without operationalising how different clinical tasks carry varying degrees of risk, trust, and need for human oversight.[15], [16], [17] LLMs are increasingly accessible to ICU clinicians and are already being used informally in intensive care settings. ICU clinicians, however, require practical frameworks that reflect the realities of bedside decision-making, proportional clinician oversight, and accountability, because these tools operate probabilistically, lack contextual awareness, and may generate plausible but incorrect outputs that can directly affect patient safety.

This review's novel contribution is the development of a clinician-led, pragmatic, risk-stratified framework tailored to Australian ICU practise. This framework maps LLM-supported tasks with the potential consequences of error, decision reversibility, and the level of clinician oversight required, thereby supporting safe, transparent, and context-appropriate integration in routine ICU workflows. This narrative review is aimed at frontline ICU clinicians and emphasises practical, risk-based applications of LLMs across clinical, educational, and administrative workflow domains, supported by a suitable governance framework consistent with emerging international guidance.^18^

## 2 Methods

We conducted a clinician-led narrative review synthesising current evidence, expert commentary, and policy literature on the use of LLMs in intensive care medicine. This approach supported conceptual integration and framework development rather than quantitative synthesis. This framework is derived from a narrative synthesis of heterogeneous literature and expert interpretation and should be considered hypothesis-generating rather than evidence-weighted.

A targeted literature search of PubMed, EMBASE, and Google Scholar identified relevant publications from January 2020 to January 2025. Search terms included combinations of ‘large language model’, ‘generative AI’, ‘artificial intelligence’, ‘ChatGPT’, ‘critical care’, ‘intensive care’, ‘clinician decision support’, ‘medical documentation’, and ‘healthcare ethics’. Reference lists of key articles were also reviewed.

Publications addressing clinically relevant applications, governance implications, or safety considerations of LLM use in critical care were included. Purely technical studies without direct clinical relevance were excluded.

The literature was synthesised thematically, grouping use cases by clinical risk, error consequences, reversibility, and required oversight. These dimensions informed the development of the clinician-led risk-stratified framework. The detailed search strategy is provided in Supplementary Appendix 3.

## 3 Large language models: key concepts relevant to safe clinical use

Large language models are AI systems designed to generate human-like text based on patterns learned from large datasets, rather than on verified factual understanding. Their outputs are probabilistic and derived from statistical associations in the training data, rather than from true comprehension or situational awareness.[18], [19], [20]

This distinction is particularly important in clinical environments. Although LLM-generated responses may appear authoritative, they may be incomplete or incorrect, particularly in high-risk ICU settings. Overreliance without verification carries the potential for clinical and medicolegal consequences.^10^^,^^11^^,^^21^

At the same time, LLMs demonstrate strengths in language-based cognitive tasks such as summarisation, explanation, and synthesis of large volumes of text, with emerging evaluations suggesting clinically meaningful performance in structured tasks when appropriately supervised.^6^^,^^22^^,^^23^ These capabilities underpin their potential utility in selected ICU workflows and reinforce the need for careful oversight, risk stratification, and clearly defined, context-appropriate use cases.

Emerging developments, including multimodal systems such as vision-language models (VLM) and concept-level architectures designed to encode higher-order representations, may expand contextual integration beyond text-based tasks. However, these approaches remain in early evaluation and have not yet been validated for high-risk ICU decision-making. Accordingly, this framework is anchored in currently deployable general-purpose language models rather than speculative architectures (Supplementary Appendix 1). Key terminology and foundational concepts are summarised in Supplementary Table S1 and S2.

## 4 Framework development and consensus process

The risk-stratified framework was developed through an iterative, structured, expert-informed process amongst the six authors, drawing on expertise across intensive care, digital health and informatics. This process followed a structured consensus approach, incorporating successive rounds of discussion, critical appraisal, and refinement until consensus was reached.

Three criteria formed the conceptual foundation for tier assignment: the potential for patient harm arising from an erroneous LLM output, the reversibility of a decision informed by that output, and the degree of clinician autonomy required to safely interpret and act upon it. These criteria were applied iteratively across candidate use cases, with alternative tier structures and framings considered before convergence on the current three-tier model. Tier boundaries were determined by clinical consequences rather than technical model characteristics, reflecting the authors’ view that governance and oversight requirements should scale with risk, irrespective of the sophistication of the underlying system.

It is acknowledged that this framework is derived from expert synthesis and structured consensus rather than prospective empirical validation. Tier assignment should therefore be regarded as clinically reasoned proposals rather than evidence-weighted determination, and the framework is intended as a hypothesis-generating foundation for future evaluation rather than a practise-ready guideline.

## 5 A pragmatic risk-stratified framework for LLM use in the ICU

Building on the core properties and limitations of LLMs outlined above, we propose a pragmatic, clinician-led, risk-stratified framework to guide their safe and context-appropriate use in intensive care medicine. Rather than focusing on technical capability or model performance, the framework is anchored in clinical risk, specifically the potential consequences of error, the reversibility of decisions informed by LLM output, and the degree of human judgment and oversight required.[24], [25], [26]

In the absence of prospective, ICU-specific trials evaluating LLM integration, tier assignment is based on a structured appraisal of task-specific harm potential, informed by clinical governance frameworks and critical care expertise.

**Fig. 1:**
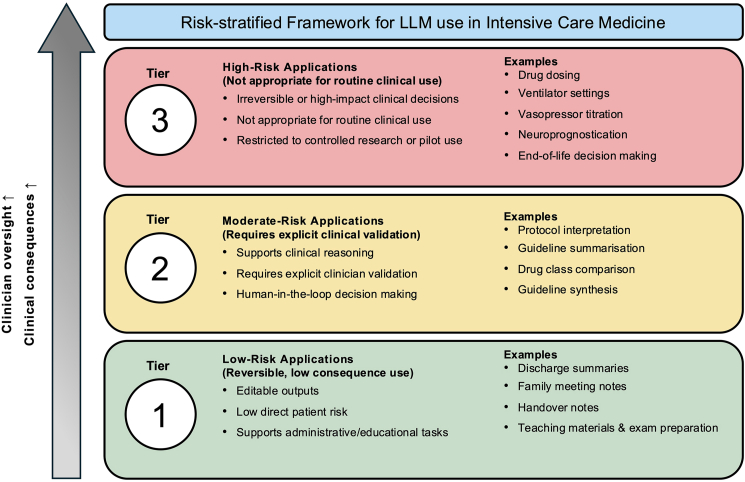
**Risk-stratified framework for large language model use in intensive care**. Applications of large language models are stratified by the potential clinical consequences of errors and the reversibility of decisions. As potential harm increases, acceptable use narrows, and the required level of clinician oversight and governance correspondingly escalates. The framework emphasises proportional human oversight rather than model sophistication.

The framework categorises LLM-supported tasks into three tiers of increasing clinical risk (Fig. 1; an expanded visual representation of the framework is provided in the Supplementary Figure). Importantly, progression across tiers is determined by clinical context and consequences, rather than by the model's sophistication.^15^^,^^27^ Identical LLM outputs may therefore be appropriate in one setting and unacceptable in another.

### 5.1 Tier 1: low-risk, reversible applications

Tier 1 comprises low-risk, high-frequency cognitive tasks in which errors are unlikely to cause direct patient harm and are readily detected and reversible. These applications are typically administrative, educational, or supportive and do not directly inform diagnostic or therapeutic decisions.^28^

Examples include drafting discharge summaries, documenting family meetings and handovers, generating teaching materials, preparing exam resources, and supporting explanations of pathophysiology. In these contexts, LLMs function primarily as time-saving cognitive aids that reduce administrative burden and support efficiency without replacing clinical judgement.

Early experience with ambient AI documentation tools, primarily used outside the ICU, indicates reduced documentation time and perceived cognitive load, with possible improvements in clinician–patient interactions. However, ICU-specific validation remains limited.

Although Tier 1 applications carry relatively low risk, outputs should remain subject to clinician review before use to ensure accuracy, contextual relevance, and alignment with local practise.^13^ At this level, informal individual use may be reasonable in low-risk contexts, provided responsibility for final clinical documentation remains clearly with the clinician.

### 5.2 Tier 2: moderate-risk applications requiring clinician oversight

Tier 2 encompasses applications in which LLMs may inform clinical reasoning, prioritisation, or planning, but must not directly guide patient management.^25^ Errors in this tier carry greater potential consequences and therefore require deliberate clinician interpretation, validation, and contextualisation.

Appropriate Tier 2 applications include guideline and protocol interpretation, synthesis of institutional or national recommendations, drug-class comparisons, checklist generation, and preparatory summarisation of complex patient information for ward rounds. In these scenarios, LLMs may assist by organising information or highlighting relevant considerations, while final decisions remain the responsibility of the treating clinician.

A defining feature of Tier 2 use is that LLM outputs are advisory rather than directive. They should be treated as information from a reference source or a junior colleague and, hence, should not be implemented without deliberate clinical scrutiny.^24^^,^^27^ As risk increases, informal individual use becomes less appropriate, and clearer institutional guidance and governance are required.

### 5.3 Tier 3: high-risk, irreversible or contraindicated applications

Tier 3 comprises applications in which incorrect reliance on LLM may result in irreversible patient harm in a time-critical ICU context, ethical breaches, or unacceptable medico-legal risk.^10^^,^^11^ These include autonomous diagnostic or therapeutic recommendations, drug dosing, ventilator settings and management, hemodynamic titration, prognostication, triage, and end-of-life decision-making.

Such tasks require nuanced physiological reasoning, longitudinal understanding of patients, and ethical judgment that extend beyond the current validated capabilities of general-purpose LLMs. At present, these applications are considered inappropriate for routine clinical use and should be actively discouraged outside rigorously governed research environments or controlled institutional pilots with formal oversight and evaluation.^13^^,^^29^

Crucially, this framework emphasises that escalation from Tier 1 to Tier 3 is driven by clinical consequence, not by model branding or technical sophistication. Governance, accountability, and human oversight must therefore increase in proportion to clinical risk.

### 5.4 Implications for institutional governance and policy development

**Fig. 2:**
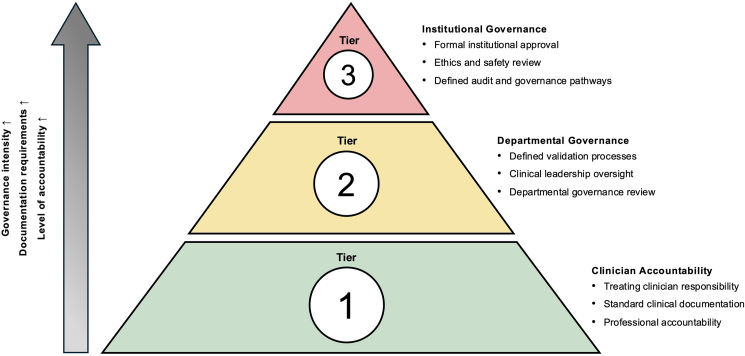
**Escalation of governance aligned with a tiered large language model application**. Accountability expands from individual clinician responsibility to departmental oversight and formal institutional governance as higher-risk applications are considered. Governance intensity, documentation requirements and oversight mechanisms increase in proportion to the tier of use.

Beyond individual clinician use, the risk-stratified framework provides a practical foundation for institutional governance, policy development, and alignment with the national digital health strategy.[30], [31], [32] This tiered structure may also support institutional decisions around acceptable use policies, access controls, and escalation pathways as LLM capabilities evolve. By aligning acceptable use with clinical risk, the framework supports proportionate escalation of governance requirements, ranging from individual accountability in Tier 1 to formal institutional oversight and evaluation in Tier 3. This approach offers health services a structured method for integrating LLMs into ICU practise while preserving clinician accountability, patient safety, and public trust (Fig. 2).

## 6 Practical applications of LLMs across ICU workflow (Table 1)

The following examples illustrate practical ICU applications of large language models, mapped explicitly to the risk-stratified framework described above. These use cases are confined to Tier 1 and Tier 2 domains, where outputs are verifiable, and clinician oversight remains central.

**Table 1:** Summary of the pragmatic risk-stratified framework for LLM Use in intensive care.

Tier	Risk Profile	Practical Applications	Ethical, Medicolegal & Governance Considerations
**1**	Low consequence; errors reversible and readily identifiable	Documentation drafting, discharge summaries, education, literature synthesis, audit planning	Individual clinician accountability; mandatory review before use; no identifiable data in public platforms
**2**	Moderate consequences; informs reasoning but does not directly determine management	Guideline synthesis, ward round case summaries, checklist generation, clinical orientation support	Explicit clinician validation; institutional guidance recommended; documentation of advisory role
**3**	High consequences; irreversible or time critical decisions	Drug dosing, ventilator adjustments, hemodynamic titration, prognostication, triage	Restricted to governed research or pilot settings; formal institutional oversight; defined accountability pathways

### 6.1 Tier 1 applications: low-risk cognitive and administrative support

#### 6.1.1 Documentation, summaries, and communication

LLMs may assist with drafting discharge summaries, handover notes, and summaries of family meetings or goals-of-care discussions.^3^^,^^33^ In this context, output functions as editable drafts, with clinicians retaining responsibility for accuracy, tone, and final content.

#### 6.1.2 Education, training, and exam support

LLMs may support ICU trainees and clinicians by summarising educational material, highlighting key concepts from guidelines or textbooks, generating questions for self-assessment and assisting with examination preparation.^3^^,^^19^ These uses are non-patient-specific and reversible.

#### 6.1.3 Research, audit, and quality improvement

LLMs may assist with literature summarisation, audit planning, data organisation, and the drafting of non-patient-facing reports, provided that identifiable patient data are excluded and outputs are verified prior to use.^13^^,^^33^

### 6.2 Tier 2 applications: clinicians-in-the-loop decision support

Tier 2 applications encompass use cases in which LLMs may support clinical reasoning, prioritisation, or preparation, but must not directly guide diagnostic or therapeutic decisions.^24^^,^^25^

#### 6.2.1 Guideline and protocol interpretation

LLMs may assist clinicians by synthesising clinical guidelines, protocols, or institutional policies to support orientation and discussion.^13^^,^^29^ Outputs should facilitate understanding rather than replace engagement with source documents, and final interpretation and decision remain the responsibility of the treating clinician.

#### 6.2.2 Clinical knowledge acquisition requiring source verification

Unlike exam-focused or self-directed learning in Tier 1, clinically applied education in Tier 2 encompasses LLM use in which outputs may directly inform a trainee's understanding of conditions, protocols, or procedures encountered in active practise. This distinction carries real risk; for example, a trainee who internalises an inaccurate LLM-generated resuscitation algorithm and applies it in an emergency setting may cause direct patient harm.

Clinician verification against authoritative sources is therefore mandatory before LLM-generated educational content informs training or bedside application.

#### 6.2.3 Ward-round preparation and complex case synthesis

In complex ICU admissions, LLMs may assist with the preparatory summarisation of patient histories, problem lists, or key investigations to support ward-round discussions.^34^ Outputs function as cognitive aids and must be verified against the clinical record.

#### 6.2.4 Checklist generation and task prioritisation

LLMs may support the generation of structured checklists or prioritisation prompts for complex care processes, aiding cognitive organisation without substituting for clinician judgement, local policy, or established clinical pathways.^25^^,^^27^ Practical use of LLMs in low and moderate-risk tiers may be supported by structured prompting strategies, as outlined in Table 2. Additional practical considerations regarding model selection, deployment, and safe prompting strategies are provided in Supplementary Appendix 2.

**Table 2:** Safe prompting domains for large language model use in the ICU.

Prompting domains	Suggested use of safe prompting
**Explicit role prompting**	• Assume the role of a senior ICU consultant, summarising… • Explain this concept at a registrar level…
**Constraining scope**	• Do not provide dosing or treatment decisions • Limit responses to educational or explanatory content
**Source anchoring**	• Base responses on established peer reviewed literature
**Force uncertainty**	• Explicitly state when evidence is weak, conflicting, or absent
**Demand structure**	• Specify response structure (e.g. headings and bullet points) • Summarise in no more than 6 bullet points

### 6.3 Tier 3 application

Tier 3 applications are considered inappropriate for routine clinical use outside rigorously governed research or controlled institutional pilot settings, as outlined above.^10^^,^^11^ These tasks include autonomous diagnostic recommendations, drug dosing, ventilator management, hemodynamic titration, prognostication, triage, and end-of-life decision-making, and require nuanced physiological reasoning, longitudinal patient understanding, and ethical judgement beyond the currently validated capabilities of general-purpose LLMs. Crucially, a fluent and confident LLM response in these domains should not be mistaken for a reliable one.

## 7 Clinical implementation of LLMs in ICU practise

### 7.1 Workflow integration into ICU practise

The tier framework maps onto the structure of a typical ICU day. The governing principle is not which task an LLM can perform, but when its outputs enter clinical thinking and under what conditions oversight can be guaranteed.

Prior to the morning ward round, Tier 2 applications are appropriate for preparatory cognitive work, such as synthesising overnight events, organising complex problem lists, or retrieving relevant guideline summaries, with all outputs verified against the clinical record before use. During and after rounds, Tier 1 applications support documentation and communication: handover notes, discharge summaries, family meeting summaries, and training and teaching materials. Research, audit, and literature tasks similarly fall within Tier 1 and may be conducted with supervised individual use.

Tier 3 tasks, including drug dosing calculations, ventilator titration, hemodynamic management, prognostication, and triage decisions, must remain outside the LLM workflow regardless of time pressure, workload, or apparent confidence in the output.

### 7.2 Responsible LLM use in ICU practise (Fig. 3, Table 3)

The boundary between tiers is determined by clinical consequence, not model sophistication or response fluency. Safe LLM use in the ICU requires deliberate clinician behaviour, not passive consumption of generated output. Clinicians should approach LLM outputs as they would those from a capable but unsupervised junior colleague: a useful starting point, but one that requires verification, contextualisation, and independent clinical judgement before action.

**Fig. 3:**
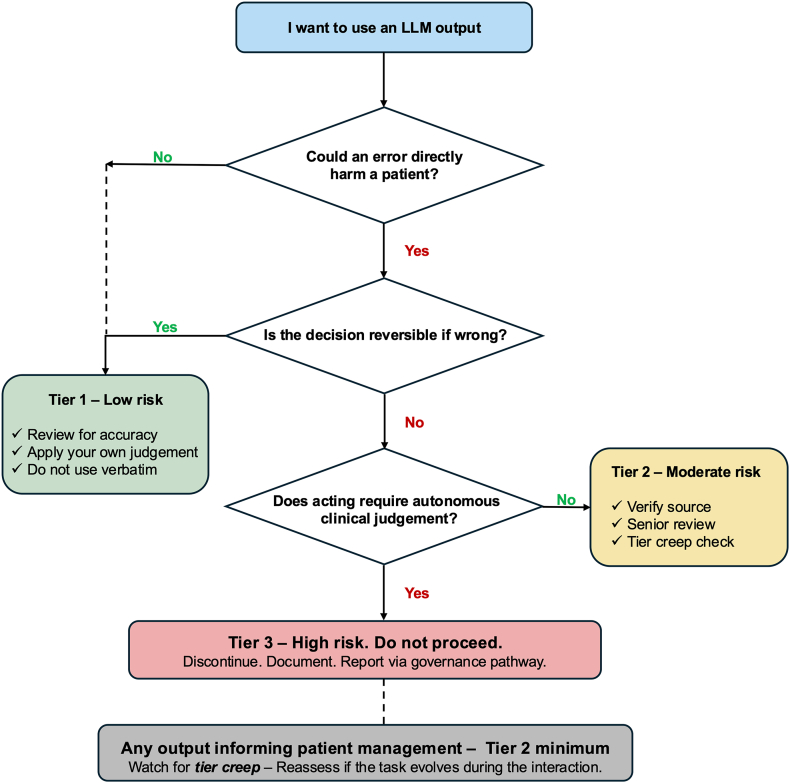
**Decision aid flowchart for risk-stratified LLM use in the intensive care unit.** Three sequential questions guide the assignment to a tier based on harm potential, reversibility, and the required level of clinician autonomy. Tier-specific verification steps apply once the tier is determined. Any output informing a patient management decision should be treated as Tier 2 minimum. LLM = Large language model; ICU = intensive care unit.

**Table 3:** LLM use decision aid for ICU clinicians.

Step	Question	Yes	No	If No – action required
**Step 1 – Tier Assignment**				
Could an error directly harm patient?		Tier 2 or 3	Tier 1 likely	Proceed to step 2
Is the decision reversible if wrong?		Tier 1 likely	Tier 2 or 3	Proceed to step 2
Does acting require autonomous clinical judgement?		Tier 3 - stop	Tier 2 or below	Do not proceed outside governed research
**Step 2 – Tier 1 verification**				
Output reviewed for accuracy and relevance?		Proceed	**–**	Review before use
Final content reflects your judgement, not verbatim LLM output?		Proceed	**–**	Revise before use
**Step 3 – Tier 2 oversight**				
Output verified against clinical record or authoritative source?		Proceed	**–**	Verify before use
Senior clinician reviewed output informing patient management?		Proceed	**–**	Escalate before acting
Still performing the same task you started? (***tier creep*** check)		Proceed	**–**	Reassess tier
Unexpected or implausible output documented and reported?		Proceed	**–**	Discontinue and report

(−) Not applicable: a ‘No’ response requires the action listed before proceeding.

Structured prompting that provides tier-level clinical context, explicitly specifies the task, and requests references for caveated responses improves output quality and reduces the risk of confident but incorrect information.

A key behavioural risk is ***tier creep***: beginning with a Tier 1 task and progressively incorporating LLM outputs into clinical reasoning without recognising the transition into Tier 2 or Tier 3 territory. Clinicians should periodically re-evaluate the nature of the task at hand and whether the level of oversight remains proportionate. Any output that informs a patient management decision should be treated as Tier 2 at a minimum, regardless of how the interaction began.

When unexpected, impossible, or potentially harmful outputs are generated, clinicians should discontinue use for those tasks, document the incident, and report through the designated departmental governance pathway.

## 8 Ethical, medicolegal and governance considerations

The ethical and medicolegal implications of using LLMs in ICUs are closely tied to the clinical context and the potential consequences of errors.^13^^,^^27^ As such, ethical risk and governance requirements should scale proportionately across the risk tiers described above, rather than being treated as uniform or abstract concerns. This approach is consistent with the Australian Commission on Safety and Quality in Health Care AI Clinical Use Guide, which emphasises clinician accountability for all AI outputs that inform clinical decisions, transparency with patients, and the need for governance proportionate to clinical risk.^17^

### 8.1 Transparency, disclosure, and documentation

Transparency about the use of LLMs is a core ethical requirement, but expectations for disclosure vary based on clinical risk.^13^ In Tier 1 applications, such as drafting documentation or providing educational support, the use of LLMs may reasonably occur without explicit patient disclosure, provided clinicians retain responsibility for accuracy and final content. As LLM outputs increasingly influence clinical reasoning and care planning (Tier 2), clearer documentation and a shared understanding within clinical teams become increasingly important.^35^ For higher-risk or institutionally deployed systems, formal policies on disclosure, consent, and documentation will likely be required as standards evolve.^30^^,^^36^^,^^37^

### 8.2 Accountability and clinician responsibility

Regardless of tier, clinical responsibility remains with the treating clinician. LLMs do not bear moral or legal accountability, and their outputs must not be treated as authoritative or directive.^10^^,^^24^ However, the responsibility shifts subtly as use moves from informal, individual clinicians’ tools (Tier 1) towards institutionally endorsed or integrated systems (Tier 2 and beyond) within Australian healthcare organisations. In these settings, health services assume increasing responsibility for governance, validation, training, and oversight, while clinicians remain accountable for how outputs are interpreted and applied in practise.^30^

### 8.3 Automation bias, trust, and cognitive authority

Ready access to fluent, confident AI-generated text introduces the risk of automation bias and overreliance, particularly in cognitively demanding ICU environments.^38^ These risks increase with task complexity and clinical consequences, reinforcing the need for explicit human-in-the-loop safeguards. Preserving clinician judgement and critical appraisal is essential to maintaining safe decision-making and professional integrity.

### 8.4 Equity and data representativeness

Many LLMs are trained predominantly on high-resource, Western datasets, raising concerns about bias and generalisability in diverse or resource-limited ICU contexts.^39^ Uncritical deployment risk amplifies existing inequities in care delivery, particularly when models are applied to populations or settings not reflected in their training data. Ethical use, therefore, requires awareness of these limitations and caution in extrapolating outputs across cultural, linguistic, or health system boundaries.

### 8.5 Professional identity of the intensivist

The integration of LLMs into ICU workflows raises legitimate questions about professional identity and the nature of clinical expertise.^27^ While LLMs may augment information processing and administrative efficiency, core elements of intensive care practise, including clinical judgment under uncertainty, ethical reasoning, procedural skill, and presence with patients and families, remain inherently human. A risk-stratified approach reinforces this distinction by positioning LLMs as supportive tools, rather than substitutes for clinical responsibility or relational care.

## 9 Limitations, risks, and safety considerations

This review has several important limitations that reflect both the evolving nature of LLMs and the methodological constraints of a clinical-led narrative synthesis. While a growing body of literature addresses AI and machine learning in critical care, prospective evaluation of LLM-specific integration in ICU settings remains limited.^23^ The framework should therefore be interpreted as clinically reasoned and hypothesis-generating rather than empirically validated.

### 9.1 Model limitations and reliability

LLMs operate probabilistically rather than deterministically and lack true clinical understanding or situational awareness of individual patients or ICU contexts.^19^^,^^20^ They are unable to independently verify factual accuracy or validate sources, necessitating clinician oversight for all outputs.^10^^,^^40^ Even in domains such as literature synthesis or evidence summarisation, LLMs should be regarded as assistive tools rather than autonomous systems.^33^ In addition, reliance on historical training data introduces risks of temporal bias and outdated information, while limited context windows constrain the integration of longitudinal patient narratives and evolving clinical states.^3^

### 9.2 Hallucination and fabrication risk

A recognised limitation of LLMs is their tendency to produce fluent yet incorrect information, often called ‘*hallucinations’* and sometimes more clinically referred to as ‘*confabulations*’, where fabricated content appears coherent and confident.^10^^,^^11^ In critical care settings, such errors may be particularly dangerous when applied to rare syndromes, complex physiology, or specialised interventions. This risk emphasises the need for verification and limits the appropriateness of LLM use beyond low- and moderate-risk applications.

### 9.3 Bias and representation issues

LLMs trained predominantly on Western-centric and high-resource datasets may inadequately represent diverse populations, low-resource ICUs, non-English speaking environments, and Indigenous and minority groups.^39^ Embedding such biases into clinical workflows risks perpetuating inequities in critical care delivery and limits the generalisability. Mitigation strategies include using diverse training datasets, conducting regular algorithmic audits for disparate impacts, and educating clinicians on bias recognition and culturally responsive practise.

### 9.4 Data privacy and confidentiality

Data governance remains a major safety challenge for LLM deployment in the ICU.^13^^,^^30^ Use of public or cloud-based interfaces increases the risk of health information leakage through prompt logging, data retention, or model retraining.^30^ Uploading identifiable clinical information may expose patients and institutions to data security breaches or re-identification risks, reinforcing the need to treat LLMs as external cognitive aids rather than integrated components of clinical systems unless robust safeguards are in place.

### 9.5 Medicolegal uncertainty

Clear medicolegal frameworks governing the use of LLMs in clinical practise are currently lacking.^12^^,^^13^^,^^27^ Responsibility for decisions informed by LLM outputs, therefore, remains with the treating clinician, compounded by limited prospective validation, poor explainability, and challenges in auditability and legal defensibility.^10^^,^^27^ These uncertainties further support a cautious, risk-stratified approach to implementation.

## 10 Conclusion

LLMs are increasingly present in the ICU, often used informally and without structured oversight. While demonstrating genuine utility in language-based cognitive tasks, their probabilistic nature and susceptibility to hallucinations pose significant risks in ICU settings. This pragmatic, clinician-led, risk-stratified framework aligns LLM-supported tasks with the consequences of errors, the reversibility of decisions, and the required clinician oversight to preserve accountability, clinical judgement, and patient safety.

This framework should be regarded as a clinically reasoned, hypothesis-generating proposal rather than an evidence-weighted guideline; it is intended as a foundation for future prospective evaluation. Responsible integration into Australian critical care will depend on governance, transparency, robust institutional oversight, and an unwavering commitment to clinician-centred decision-making.

## Ethics approval and consent to participate

Not applicable.

## Consent for publication

Not applicable.

## Availability of data and material

Not applicable.

## CRediT authorship contribution statement

**Nilesh Anand Devanand:** Conceptualisation, Writing – Original Draft, Writing – Review & Editing, Project administration. **Santosh Verghese** Conceptualisation, Methodology, Writing – Review & Editing. **Stephen Bacchi** Conceptualisation, Methodology, Writing – Review & Editing. **Christopher Bain** Writing – Review & Editing. **Ashwin Subramaniam** Conceptualisation, Methodology, Writing – Review & Editing. **Krishnaswamy Sundararajan:** Conceptualisation, Methodology, Writing – Review & Editing, Supervision, Project administration.

## Use of artificial intelligence in manuscript preparation

Artificial intelligence-based tools were used during the preparation of this manuscript to assist with language refinement, structural organisation, and editorial clarity. No artificial intelligence system was used to generate original scientific content, interpret data, or make clinical judgements. All content was reviewed, verified, and approved by the authors, who take full responsibility for the accuracy and integrity of the manuscript.

## Funding

This study received no funding support.

## Conflict of interest

The authors declare the following financial interests/personal relationships which may be considered as potential competing interests: Ashwin Subramaniam (AS) is an Associate Editor and Editorial Board Member for Critical Care and Resuscitation and a sub-specialty editor for the Internal Medicine Journal. In accordance with journal policy, he had no involvement in the peer review or editorial decision-making for this manuscript and had no access to information regarding its review. Full responsibility for the editorial process was delegated to another editor.

All other authors declare no competing interests. If there are other authors, they declare that they have no known competing financial interests or personal relationships that could have appeared to influence the work reported in this article.
