# Supplementary material for: A pragmatic risk-stratified framework for using large language models in intensive care medicine: A narrative review
Source: Crit Care Resusc. 2026 Jun 25;28(3):100194. doi: 10.1016/j.ccrj.2026.100194 (PMC13318555; doi:10.1016/j.ccrj.2026.100194)
Supplement: Multimedia component 1 [file mmc1.docx]

**Supplementary Appendix 1: Technical overview of large language models**

This appendix provides a technical overview of large language model architectures and behaviour, offering additional context beyond the clinical focus of the main text.

LLMs are a category of machine learning systems that analyse relationships between words and phrases ^20^  using advanced natural language processing (NLP) techniques. They operate by converting text into numerical representations (tokens) and generating outputs based on learned statistical associations, rather than on verified factual understanding.

As a result, LLM outputs may appear fluent and authoritative while lacking true comprehension or situational awareness. This probabilistic behaviour is particularly relevant in high-risk clinical environments such as the ICU, where apparent coherence should not be equated with accuracy or reliability.

Despite these limitations, LLMs perform well in language-based cognitive tasks, including summarisation, explanation, and synthesis of large volumes of text. These properties underpin their potential utility as assistive tools in selected ICU workflows, while reinforcing the need for careful oversight, risk stratification, and clearly defined use cases.

**Supplementary Appendix 2: Practical considerations for LLM use in the ICU**

**Prompt engineering and safety verification for clinicians**

The quality and appropriateness of LLM outputs are influenced by how clinicians interact with these systems. Prompts should be designed to support cognitive assistance rather than replace clinical reasoning or decision-making. Outputs should be treated as provisional and subject to verification prior to use. Developing simple, consistent verification habits may help clinicians establish safer patterns of day-to-day use.

**Model-specific considerations for ICU use**

Given the rapid evolution of LLM architecture and deployment platforms, recommending a single preferred system for ICU use is neither feasible nor appropriate. Instead, ICU clinicians and institutions should focus on class-level characteristics that influence safety, governance, and suitability within high-risk ICU environments, including data handling, transparency, and auditability.

**General-purpose cloud-based LLMs**

General-purpose, cloud-based LLMs are designed for broad language tasks and are best suited to low-risk cognitive activities such as education, concept explanation, and drafting non-clinical documentation. Limitations include variable factual precision, a risk of hallucination, limited transparency about training data, and uncertainty about data retention. These models should not be used for patient-specific decision making or for processing identifiable clinical information in routine practise.

**Medical domain language models**

These models, trained on biomedical literature or clinical datasets, may demonstrate improved domain specificity in controlled settings. However, benchmark performance does not equate to clinical safety, and real-world validation in ICU environments remains limited. Their use should therefore be restricted to educational, research, or clinician-supervised decision support contexts.

**Open-Source and On-Premise LLMs**

These models offer advantages in data sovereignty and local governance but require mature institutional infrastructure. Validation, monitoring, cybersecurity, and risk mitigation processes are essential prerequisites for deployment. Adoption should be driven by organisational readiness rather than perceived technical performance.

**Deployment considerations (cloud vs on-premise)**

Regardless of model class or deployment strategy, LLMs used in the ICU should follow a risk-stratified clinical framework with human oversight proportional to the potential clinical consequences of error. At present, routine use should prioritise low-risk cognitive support tasks, with increasing restriction and scrutiny as clinical risk increases.

**Supplementary Appendix 3: Literature & evidence search strategy**

**Review approach**

This manuscript was informed by a clinician-led narrative review undertaken to support framework development rather than exhaustive evidence synthesis or quantitative pooling. The intent of the search strategy was to identify representative clinical, methodological, ethical, and policy literature relevant to the use of LLM in intensive care and other high-risk clinical environments.

**Databases and time frame**

A targeted literature search was conducted across the following databases: PubMed, EMBASE, and Google Scholar. The search covered publications from January 2020 to January 2025, reflecting the period during which generative artificial intelligence and LLMs emerged in clinical contexts.

**Search terms**

Search terms were used in combination and included: ‘large language model’, ‘generative AI’, ‘artificial intelligence’, ‘ChatGPT’, ‘clinical decision support’, ‘critical care’, ‘intensive care’, ‘ICU’, ‘medical documentation’, ‘clinical workflow’, ‘healthcare ethics’, ‘governance’. The reference list of key articles was hand-searched to identify additional relevant publications.

**Scope of included literature**

The literature informing this review included peer-reviewed clinical studies and narrative reviews; methodological and conceptual analyses of LLMs in healthcare; policy documents, position statements, and regulatory commentary; and ethical, medico-legal, and governance-focused publications relevant to high-risk clinical settings. Grey literature and expert commentary were included where relevant to governance, implementation, or ethical considerations. Purely technical or model-development studies without clear relevance to clinical use were excluded.

**Evidence synthesis**

Identified sources were synthesised thematically, with emphasis on clinical context and the consequences of error; the reversibility of decisions informed by LLM outputs; the degree of clinician oversight required; and the ethical, medico-legal, and governance implications. This synthesis informed the development of a pragmatic, risk-stratified framework for using LLMs in the ICU.

**Methodological limitations**

As a narrative review, this approach does not aim to provide an exhaustive or systematic assessment of all available literature, nor does it formally assess study quality or risk of bias. However, this methodology was selected to enable integration of heterogeneous evidence sources and clinical judgement in an area characterised by rapid technological evolution and limited prospective validation.

**Supplementary Figures and Table**

**Table S1. Essential terminology related to the pragmatic use of artificial intelligence (AI) and large language models (LLMs).**

| **Terminology** | **Definition** |
| --- | --- |
| **Artificial Intelligence (AI)** | A broad field of computing focused on systems capable of performing tasks that require human cognitive functions, such as reasoning, pattern recognition, and decision support. In healthcare, AI encompasses a range of tools designed to augment, rather than replace, clinical judgement. |
| **Machine Learning (ML)** | A subset of AI in which systems learn patterns from data to improve task performance without being explicitly programmed. In clinical contexts, ML is commonly used for prediction, classification, and pattern recognition. |
| **Large Language Models (LLM)** | A class of ML models trained on an extensive collection of text to generate and interpret human language. LLMs are neural network architectures to produce contextually relevant text and are primarily applied to language-based tasks such as summarisation, explanation, and information synthesis. |

**Table S2. The basics of large language models**

| **Terminology** | **Definition** |
| --- | --- |
| **Embedding** | The process by which words or phrases are converted into numerical representations that allow language models to recognise patterns and relationships within text. |
| **Words and Tokens** | Words are units of language used by humans. Tokens are the smaller text units used by language models to process and generate language, which may represent whole words, subwords, or characters. |
| **Encoding and Decoding** | Encoding refers to the conversion of text into tokens that a model can process.  Decoding refers to the generation of human-readable text from these tokens. |
| **Natural Language Processing** | A field of AI focused on enabling computers to analyse, interpret, and generate human language in a structured and meaningful way. |
| **Transformer Encoder-Decoder** | The underlying model structure of most modern language models enables them to process contextual relationships within text and generate coherent language. |

**Supplementary Figure: A risk-stratified framework for safe use of LLMs in the ICU**
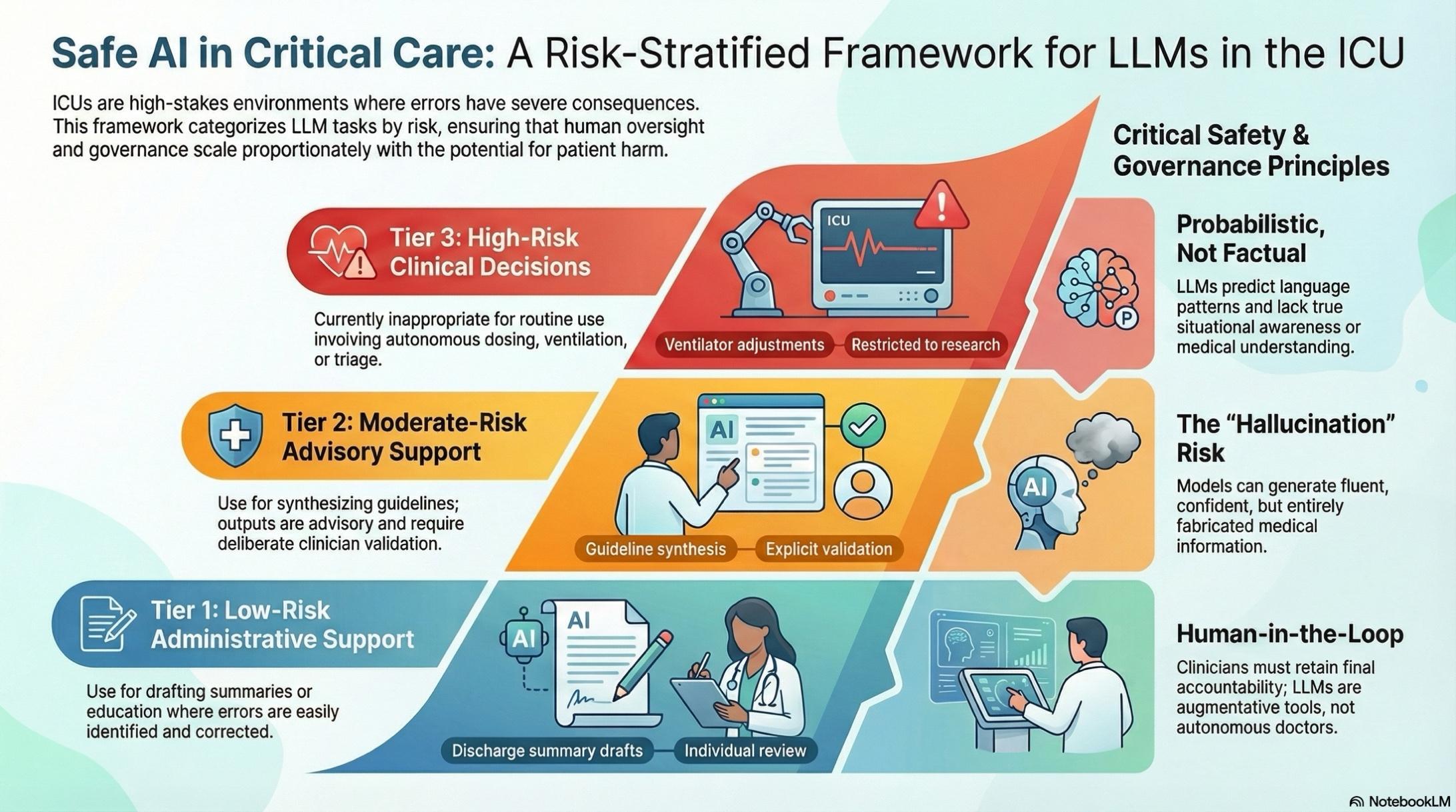


**Supplementary Figure. Risk-stratified framework for large language models in intensive care**

Applications are categorised by clinical consequences and reversibility of the decision. Tier 1 comprises low-risk administrative tasks subject to clinician review. Tier 2 includes an advisory function requiring explicit clinician validation. Tier 3 represents high-risk clinical decisions that are currently inappropriate for routine use outside of governed research settings. Governance intensity and accountability increase in proportion to clinical risk. LLM = Large Language Model. ICU = Intensive Care. Figure generated using NotebookLM (Google LLC, Mountain View, CA, USA;2024).
